# Supplementary material for: Diabetic and Hypertensive Disorders Following Miscarriage: A Protocol for Systematic Review and Meta-Analysis
Source: Int J Environ Res Public Health. 2022 Jul 7;19(14):8324. doi: 10.3390/ijerph19148324 (PMC9318385; doi:10.3390/ijerph19148324)
Supplement: Supplementary file 1 [file ijerph-19-08324-s001.zip › ijerph-1791957-supplementary.pdf]

Table S1: Search Strategy for Ovid/MEDLINE

#	Search terms
1	exp "Abortion, Spontaneous"/ or exp "Abortion, Induced"/ or exp "Fetal Death"/
2	("Miscarriage" or "Habitual miscarriage" or "Recurrent miscarriage" or "Spontaneous abortion" or "Induced abortion" or "Habitual abortion" or "Recurrent abortion" or "Incomplete Abortion" or "Spontaneous loss" or "Pregnancy loss" or "Infant loss" or "Fetal loss" or "Foetal loss" or "Fetus loss" or "Foetus loss" or "Fetal death" or "Foetal death" or "Fetus death" or "Foetus death" or "Fetal demise" or "Foetal demise" or "Fetus demise" or "Foetus demise").ti,ab.
3	1 or 2
4	exp "Diabetes Mellitus"/
5	("Diabetes" or "Diabetes mellitus" or "Type 1 diabetes" or "Type-1 diabetes" or "Type 1 diabetes mellitus" or "Type-1 diabetes mellitus" or "Type 2 diabetes" or "Type-2 diabetes" or "Type 2 diabetes mellitus" or "Type-2 diabetes mellitus").ti,ab.
6	4 or 5
7	exp "Diabetes, Gestational"/ or exp "Pregnancy in Diabetics"/
8	(("Diabetes" or "Diabetes mellitus") adj3 ("Gestation*" or "Pregnan*")).ti,ab.
9	7 or 8
10	exp "Hypertension"/
11	("Hyperten*" or (("High" or "Increas*" or "Elevat*") adj3 "Blood pressure")).ti,ab.
12	10 or 11
13	exp "Hypertension, Pregnancy-Induced"/
14	((("Hyperten*" or (("High" or "Increas*" or "Elevat*") adj3 "Blood pressure")) adj3 ("Gestation*" or "Pregnan*")) or "Eclampsia" or "Preeclampsia" or "Pre-eclampsia" or "Pre eclampsia").ti,ab.
15	13 or 14
16	6 or 9 or 12 or 15
17	3 and 16
